# Optimal Identification of Semi-Rigid Domains in Macromolecules from Molecular Dynamics Simulation

**DOI:** 10.1371/journal.pone.0010491

**Published:** 2010-05-13

**Authors:** Stefan Bernhard, Frank Noé

**Affiliations:** Free University Berlin, DFG Research Center MATHEON, Berlin, Germany; Massachusetts Institute of Technology, United States of America

## Abstract

Biological function relies on the fact that biomolecules can switch between different conformations and aggregation states. Such transitions involve a rearrangement of parts of the biomolecules involved that act as dynamic domains. The reliable identification of such domains is thus a key problem in biophysics. In this work we present a method to identify semi-rigid domains based on dynamical data that can be obtained from molecular dynamics simulations or experiments. To this end the average inter-atomic distance-deviations are computed. The resulting matrix is then clustered by a constrained quadratic optimization problem. The reliability and performance of the method are demonstrated for two artificial peptides. Furthermore we correlate the mechanical properties with biological malfunction in three variants of amyloidogenic transthyretin protein, where the method reveals that a pathological mutation destabilizes the natural dimer structure of the protein. Finally the method is used to identify functional domains of the GroEL-GroES chaperone, thus illustrating the efficiency of the method for large biomolecular machines.

## Introduction

The mechanical properties of biomolecules and their complexes are essential to molecular function, because many molecular processes are accompanied by conformational changes, in which domains of the molecule must be able to move with respect to each other [1]–[5]. For example the mechanical properties of actin are strongly coupled to polymer formation and degradation [6]. Such a coupling between different functional states and aggregation states of molecules and their mechanical properties are ubiquitous in biology. Understanding the nanomechanics of the biomolecules, i.e. the semi-rigid domains and their relative mobility for each given conformational or aggregation state, is thus one of the key questions in molecular biophysics allowing for both (i) the understanding/analysis of the molecular nanomechanics and (ii) paving the ground for efficient large-scale coarse-grained simulations [7]–[9].

The first step to analysis and simulation of molecular nanomechanics is the identification of the rigid and flexible parts of biomolecules in different chemical, conformational or aggregate states considered. Conventional experimental techniques, like for example nuclear magnetic resonance (NMR), provide limited information about these processes.

One approach to identify the rigid and flexible parts in biomolecules is to partition the system into domains (also called “groups” or “clusters” in other works) that are nearly rigid. In the coarse-grained model, these domains can only move as a rigid body with six degrees of freedom (3 translation +3 rotation). Such a low dimensional model of the original high-dimensional dynamics yields itself easily to the understanding of essential mechanical properties of the molecule and how they change between conformations. Clearly, such a model only approximates the real mobility and the approximation error will depend on the number of domains considered and on the flexibility/rigidity of the molecule in the conformation considered. Consequently, such a model is better suited for describing functional transitions or aggregation than for processes involving much flexibility, such as folding.

Several methods for the identification of nearly rigid domains in biomolecules have been proposed that produce similar but not identical results. They can be categorized into model-based methods, where structural aspects such as hydrophobicity, topology, structural homology or for e.g. identical sequence motifs serve to identify the smallest building blocks [10]–[13]. In this category there are also a variety of methods that try to optimize certain structural properties of protein domains, such as the distance-mapping [14], interface area [15], specific volume [16] and compactness of the domain [17]. In [18] a cluster method is proposed that uses contact measures and fuzzy logic to define protein domains.

Data-based approaches in contrast define domains based on data of the flexibility of the biomolecule, such as MD simulations [19], [20]. One approach to obtain correlated motion of atoms within the molecule is (quasi) harmonic analysis, namely Principal Component Analysis (PCA) and Normal Mode Analysis (NMA) [21], [22]. Here, the motions that contribute most to the variation between the molecular configurations are described by the dominant eigenmodes of the covariance matrix or the Hessian of the potential, respectively. The subspace of the first few eigenmodes contains most of the flexibility and a number of methods have been developed to use this information in order to identify domains [23]–[25]. Other data-based approaches are based on dynamical clustering [26], hierarchical clustering of correlation patterns (HCCP) [27], and the hinge detection algorithm [19], [28]. The latter algorithm assumes that collections of atoms move as rigid bodies connected by hinges or axes of rotation. Recently, [29] has proposed a optimal method to decompose proteins into rigid domains using equilibrium fluctuations of inter-residue distances.

Normal-mode-based techniques are limited by the fact that they only use local information of the energy landscape. PCA-based clustering methods do not suffer from this limitation, but still require all structures to be fitted to a mean or reference structure before calculating the covariance matrix. Such a fitting procedure works well as long as the structures are very similar, but if very large conformational changes are involved, then structures which are very similar to each other but very different from the reference structure may become very different after the fitting and thus produce a misleading covariance matrix. Thus, it is desirable to use a method that works with internal coordinates only. Moreover, there is a lack of the domain identification techniques that avoid ad-hoc assumptions and parameter choices that indirectly influence the number of clusters. It would be rather desirable to have an explicit control of the clustering error by adjusting the number of domains, or to have the method select the number of domains such that the clustering error is below a certain threshold.

The proposed method works by defining (i) a distance-deviation matrix between atoms based on dynamical data, (ii) formulating the clustering problem as a quadratic optimization problem that is based on this matrix and (iii) solving this clustering problem to optimality and obtaining an assignment of atoms to clusters. To illustrate the strengths and limitations of this approach a number of example systems are considered: two artificial peptides Ala_5_ and 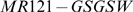 and the two biomolecules transthyretin and the chaperone complex GroEL-GroES.

The immediate use of the method is to understand dynamic processes in large macromolecules and their complexes which involve changes of molecular rigidity. This includes processes like conformational changes, ligand binding and protein aggregation [30]–[32]. Besides this, the outcome of the method can be used in a number of other biophysical problems, including the coarse-grained simulation of macromolecular encounters and association.

## Materials and Methods

The principal objective of this work is to develop a new coarse-graining technique to partition large molecular systems *optimally* into semi-rigid domains, thus providing a simple model of molecular nanomechanics. The proposed method is data-based and meets the following requirements:

1. Optimal and unique molecular partitioning for given data and number of domains
2. Works with internal coordinates only and is thus independent of a reference structure
3. Can be applied to characterize models with multiple conformations without “overlooking” rarely populated conformations
4. Error measure for coarse-grain quality and ability to adjust the accuracy by the number of domains or the maximum acceptable clustering error
5. Simple applicability and robustness - no parameters other than number of domains
6. Model independent, so that experimental findings are easily incorporated
7. Efficient and simple implementation

### Molecular rigidity and distance deviation

Inter atomic distance-deviation is a common metric used for the identification of rigid domains in proteins [33], [34]: Within a rigid domain, the euclidean distance between pairs of atoms remains constant, while it fluctuates for atom pairs that lie in different rigid domains moving relative to each other.

The analysis of local molecular rigidity is based on the distance deviation matrix 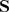, whose elements 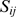 are the Euclidean distance deviations, between the atoms 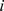 and 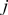 in the molecule, defined as:
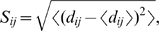
(1)where 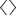 indicates the ensemble average and 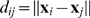 is the Euclidean distance between the atomic positions 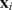 and 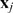. 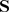 is symmetric (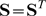), but not necessarily positive definite, it has dimensions 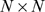 for an 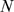 atomic molecule. In practice, the ensemble average Eq. 1 may be estimated via a time expectation value from a molecular dynamics simulation. Of course, the reliability of the estimate and thus the result of our method will depend on the length of the simulation: Only if all relevant conformations of the molecule have been visited with a probability according to the Boltzmann distribution, will Eq. 1 converge. The distance-deviation can be computed for all solute atom pairs, or for a reduced set of representative atoms, such as 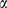-carbon atoms in order to reduce memory consumption when analyzing large macromolecules. We note that it is possible to use the matrix of squared distance deviations instead of using distance deviations. Alternatively to using simulations, Eq. 1 can be computed from realizations of an NMR ensemble or several x-ray structures of the same molecule. The mean row value of 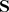 is a measure for the flexibility of individual atoms.

### Cluster membership probability

Most methods in the literature [19], [27], [28] assume that each atom is uniquely assigned to one domain. This results in a so called integer optimization problem, which is very hard to solve [35]. Reference [36] has suggested using a fuzzy membership, where formally each atom 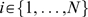 may participate in different domains 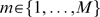 with a certain membership probability 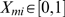. 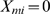 means that the motion of the atom is independent of the motion of the domain, and 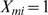 means they are perfectly synchronized. A natural normalization condition for 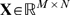 is that the total membership probability sums up to one,
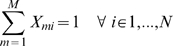
(2)As a direct consequence we can write the probability 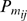 of finding the atoms 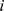 and 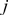 within the same domain 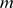 as
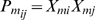
(3)As it will turn out, the optimal grouping into domains is always unique in practice (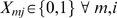). Nevertheless, the introduction of the fuzzy memberships is essential as it allows the clustering problem to be formulated as continuous quadratic optimization problem, which, in contrast to integer optimization problems can be solved efficiently for very large systems.

### Optimization problem for identifying semi-rigid domains

We define the optimal partition of the molecule into domains as the one that minimized deviations within the domains:
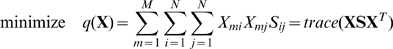
(4)This objective function measures the error describing the amount of distance deviations neglected by confining the motion of the atoms within their domains. Since a partitioning using 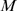 domains can always realize a 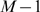 domain partitioning as a special case, increasing the number of domains relaxes the optimization problem and the optimal error is thus monotonically decreasing (see also section “MR121-GSGSW peptide”), for 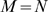, the solution 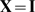 and 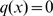 is obtained.

In contrast to heuristic coarse-graining methods the minimization problem in Eq. 4 leads to an optimal partitioning of the molecule according to the number of domains chosen. Furthermore the partitioning has no bias towards equally-sized domains, i.e. it allows for domains of very different sizes if this is requested by the structure of 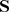.

The minimization problem in Eq. 4 together with the normalisation condition in Eq. 2 can be written into a standard quadratic optimization problem with linear constraints that is solved here in order to identify the optimal partitioning into domains.
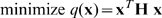
(5)

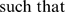


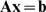


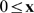
Here 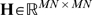 is the symmetric Hessian matrix,
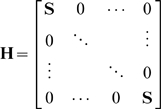
(6)and 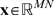 is a column vector containing the membership probabilities
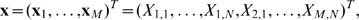
(7)with 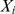 being the i-th row of 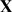. The constraint matrix 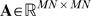 and the column vector 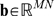 represent the equality constraints on 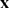. According to Eq. 2
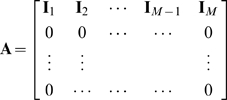
(8)where 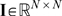 is the identity matrix, and
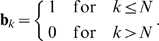
(9)


Because the Hessian matrix 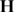 is just a composition of sub-matrix 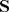, one may reduce problem size by introducing the sub-vectors, 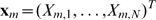 for each domain and reassemble 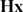 from the products 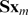. The numerical implementation is described in section “Numerical implementation”.

### Numerical implementation

The present quadratic optimization problem is solved using an active set method similar to that of Gill et al., described in [37]. The solution procedure involves two phases: the first phase involves the calculation of a feasible point 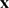 (if one exists), the second phase involves the generation of an iterative sequence of feasible points that converge to the solution.

Besides the sparse definition of 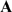 and 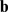 one may reduce the size of the problem from one 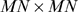-dimensional problem to 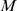 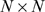-dimensional problems. Because 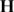 is block diagonal, one may compute 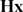 as the piecewise product 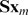 and reconstruct the vector
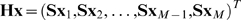
(10)in a subsequent computation. This modification reduces the memory consumption significantly (by a factor of 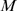), because instead of 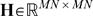 only 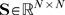 has to be held in the memory. The involved increase of computation time is insignificant. With this modification the problem size solvable on desktop computers is up to 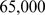 particles. We note that in large molecular systems these particles may be chosen to be backbone or 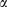-carbon atoms, so that the number of atoms of the molecule can be much larger.

### Initial condition and “successive restart”

Even though the method is robust for low 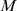 (see section “MR121-GSGSW peptide”) it was found that for larger 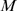 the solution depends on the initial condition (IC) that is provided to the solver. A permutation of the domains only modifies the labels and not the grouping, thus there exist at least 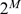 equivalent solutions. Unfortunately, there are also multiple non equivalent local minima where the solver may get trapped. In order to avoid being trapped in a bad local minimum it is advisable to choose a good initial condition 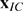.

One approach to escape from local minima is applying stochastic methods such as Monte Carlo sampling, simulated annealing or genetic algorithms. Another simple approach that has shown to work well in practice is to use the solution obtained for 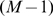 domains to construct 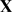 for 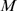 domains. This heuristic approach may done by identifying the cluster membership subvector, 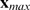, that has the maximum average contribution to 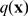 per member. Formally this is expressed by
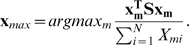
(11)The memberships within the subvector 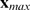 are distributed over two domains by substituting 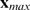 by 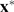, with elements 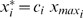 and appending 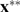 with elements 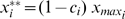 as 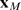. Here 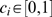 is either deterministically or randomly chosen for every atom in 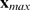. To assure that the number of atoms is conserved the sum of the membership probabilities over 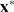 and 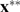 is one for all member atoms.
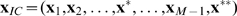
(12)This procedure assures that the clustering error is monotonically decreasing with increasing number of domains (see section “MR121-GSGSW peptide”).

### Clustering quality and number of domains

The error can be used as tool to choose the number of domains 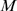, either by prescribing a desired 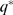 and asking for the smallest number of domains with 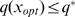, or by looking for gaps in the error series and choosing 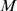 such that 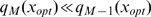. In some applications it may be desirable to have the number of domains selected automatically rather than by the user. One possible method is to select the number of domains such that the clustering error stays below a user-imposed bound, 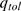. For this, it is useful to define the normalized clustering error as:
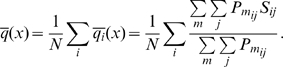
(13)Here, 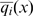 is the normalized error for atom 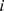. Eq. 13 is not what is optimized here, but a measure for the mean distance deviations of pairs within domains for a given number of clusters. It therefore has a direct physical interpretation and measures the quality of a clustering of the molecule into semi-rigid domains. Alternatively, Eq. 13 can be modified to use the matrix of squared distance deviations, leading to the RMSD as error measure. 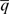 is identically zero when the molecule consists of 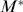 perfectly rigid domains and 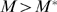 is used. 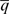 is also useful in order to make an automated choice of 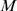: It can be set to a value the user considers as small enough, such as 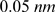. Based on this rationale, the optimal clustering is chosen by the following algorithm:

1. Compute distance-deviation matrix, 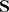

2. Set 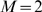

3. Compute optimal clustering 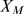 of based on 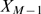.
4. If 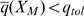 return 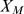

  Else 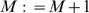, Go to 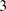.

### Computational performance

The computational performance of the method was demonstrated for the examples discussed in the “Results” section. From Table 1 is seen that the method is very efficient even for a large number of particles/domains.

**Table 1:** Computation time for selected molecular systems.

System	no. Atoms	time in seconds		
		M = 2	M = 5	M = 81
MR121-GSGSW	81	0.015	0.12	14.46
Transthyretin	229 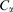	0.048	0.94	94.72
Transthyretin	2257	1.32	92.34	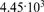
GroEL-GroES	8015 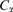	181.77	1537.01	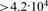

Computation time for selected molecular systems with 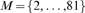 domains. Computations were done on a usual desktop computer with CPU@2.5 GHz and 6.5 GB Ram, time is given in seconds.

### Molecular models and simulation setup

To demonstrate the performance and usefulness of the method we have applied it to a series of molecular systems:

1. A 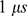 MD trajectory of Ala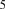, containing 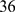 solute atoms.
2. A 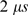 MD trajectory of the artificial peptide MR121-GSGSW [38] (i.e. a chromophore MR121 is connected with GLY-SER-GLY-SER-TRP), containing 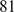 solute atoms.

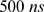
 MD trajectories of the wild type of transthyretin (PDB ID code, 1DVQ) [39], containing 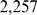 solute atoms, and two point variants 58Arg, 58His, containing 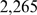 and 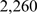 solute atoms respectively. The point mutants were generated by Modeller Release 9v5 [40].
4. A 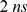 MD trajectory of the chaperone GroEL-GroES (PDB ID code, 1GRU), containing 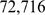 solute atoms.

All molecular dynamics trajectories were generated by the molecular dynamics package Gromacs 3.3 [41] using the standard distribution force field GROMOS96 43a2. The solutes were solvated in SPC216 water in a cubic box with at least 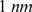 of water on each side of the solute. The structures were equilibrated with a 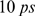 molecular dynamics simulation constraints on all bonds of the protein. A subsequent energy minimisation without position restraints was performed with a steepest descent minimization. The production runs were done with LINCS constraints [42] on the hydrogen bond length and a 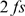 time step, the trajectory was written every 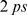. The electrostatic interactions were computed using the smooth Particle Mesh Ewald algorithm (PME), where the full direct and reciprocal space parts were calculated each step with a lattice spacing of 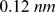. The Van der Waals interactions were computed with a cut-off at 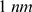. All simulations were performed with Berendsen temperature coupling and isotropic pressure coupling to 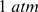. The temperatures used were 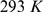 for systems 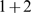 and 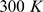 for systems 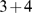.

## Results

We illustrate our approach on a number of test systems. In all cases the distance deviation matrix 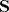 was computed from the data and the optimization problem in Eq. 5 was solved for a series of consecutive domains numbers 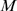 using the successive restart approach described in the “Materials and Methods” section.

### Application to Small Model Systems

#### Numerical example

Interestingly, although the method formally allows for fuzzy memberships, the optimal assignment of atoms to domains is always unique in practice, thus obtaining an exact partitioning of atoms into domains. For example, consider a hypothetical 3-atom system with the distance-deviation matrix
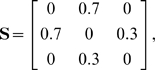
(14)which has the optimal solution for M = 2 subunits:
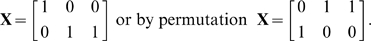
(15)Here atom 1 is placed in the first and atoms 2 and 3 are placed in the second domain. As described in Section “Cluster membership probability” the elements of the membership matrix either converge to one or to zero, i.e. the atoms tilt over to the domain that produces the smallest clustering error when including this atom. In other words, the clustering error is minimal when each atom is fully assigned to the subunit it belongs to most.

Now consider the case,
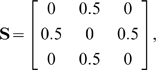
(16)that has the solution for 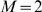


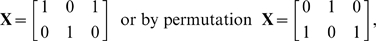
(17)with total error 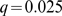. In contrast the fuzzy solution
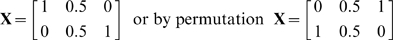
(18)has a higher total error of 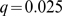. Finally, consider the pathological case of an off-diagonally uniform distance matrix which represents entirely uncorrelated motion
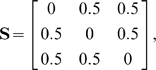
(19)which may be found for gas particles. In this case the solution for 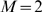 is degenerated:
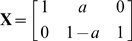
(20)In this case the total error of 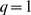 was found for all 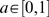. According to the uniformity of 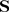 this degenerate case is never found in practice for macromolecules. Even nearly unstructured proteins will have some structure in 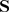 because of their bonding topology and minor deviations in 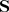 from the uniform case will cause the atoms to be uniquely assigned to one domain such that the error is minimum.

We conclude that no fuzzy memberships are found in macromolecules. Note, however, that the introduction of a fuzzy membership was still essential, because using this formulation we could express the optimization problem as a continuous quadratic optimization problem. The solution to this kind of problem is much easier than the solution to the integer optimization problem emerging by the priori assumption that the memberships must be integer values.

#### Polyalanine

**Figure 1:**
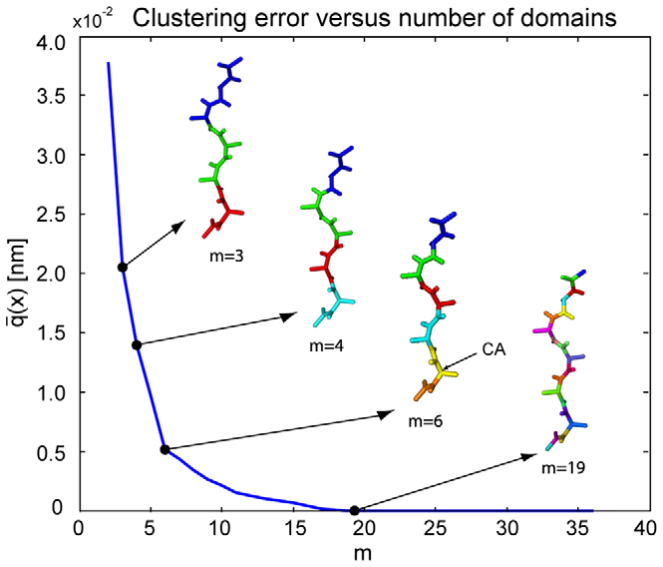
Clustering error of Ala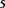 for 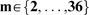 and corresponding coarse-grain structures for 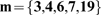 domains. The decrement of the clustering error is very steep for 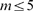 and relatively flat afterwards, suggesting that 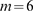 is a good choice for the number of domains (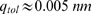). The molecule is partitioned into its four peptide planes and two end groups containing the C- and N-terminus respectively.

As a first example the optimization method was applied to Ala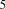 in order to demonstrate that the method can identify meaningful domains. Some of the resulting coarse-grain structures and the clustering error for 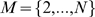 are shown in Figure 1. The sub-structures are approximately equally sized and represent the optimal partitioning for a given number of domains. As the number of domains is increased the size of the domains diminishes. For 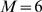, the method successfully identifies the domains that are nearly rigid due to bond angle, angle, improper dihedral and 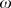-angle constraints: There are 4 domains containing the 4 peptide planes including the first but excluding the second 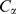 plus the 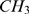 side chain. The remaining two domains contain the N-terminal and the C-terminal (see Figure 1). The small remaining clustering error reflects the vibrations still allowed within the domains, mainly due to flexibility in the improper and 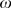-dihedrals. For 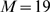 the method clusters the system into domains containing one backbone atom each along with the one side chain atom connected to it. Finally the method is shown to be consistent in the limit, because for 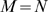 every atom is placed into a single domain (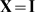), and the error is zero 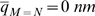 (not shown as structure).

#### MR121-GSGSW peptide

**Figure 2:**
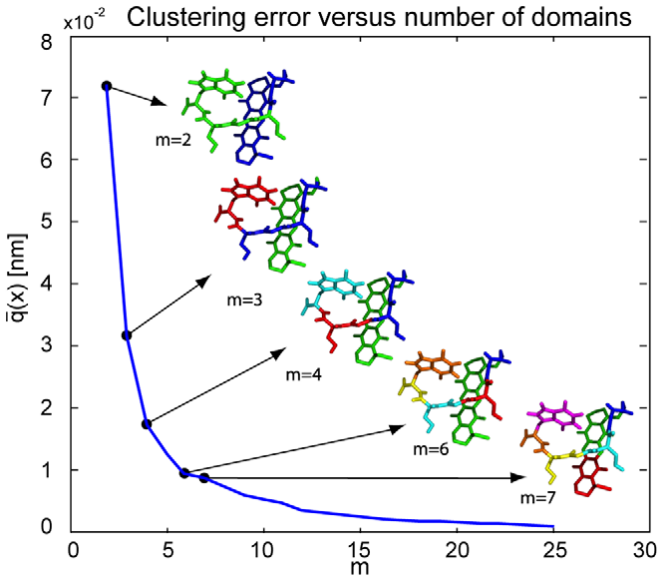
Clustering error of MR121-GSGSW for 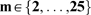 and corresponding coarse-grain structures for 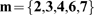 domains. The decrement of the clustering error is very steep for 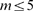 and relatively flat afterwards. For 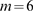 the number of domains is well balanced with the expected error (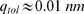).

In order to study a more complex system, the method was applied to the MR121-GSGSW peptide. Figure 2 shows a series of molecular coarse-grain structures for selected numbers of domains 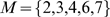. This series shows clearly how the flexible parts of the molecule subdivide into finer domains. 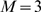 separates the GSGS chain and the chromophores, 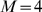 splits also the GS domains. Using more domains accounts for smaller decrease of the error until 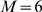 the system is split into individual residues.

**Figure 3:**
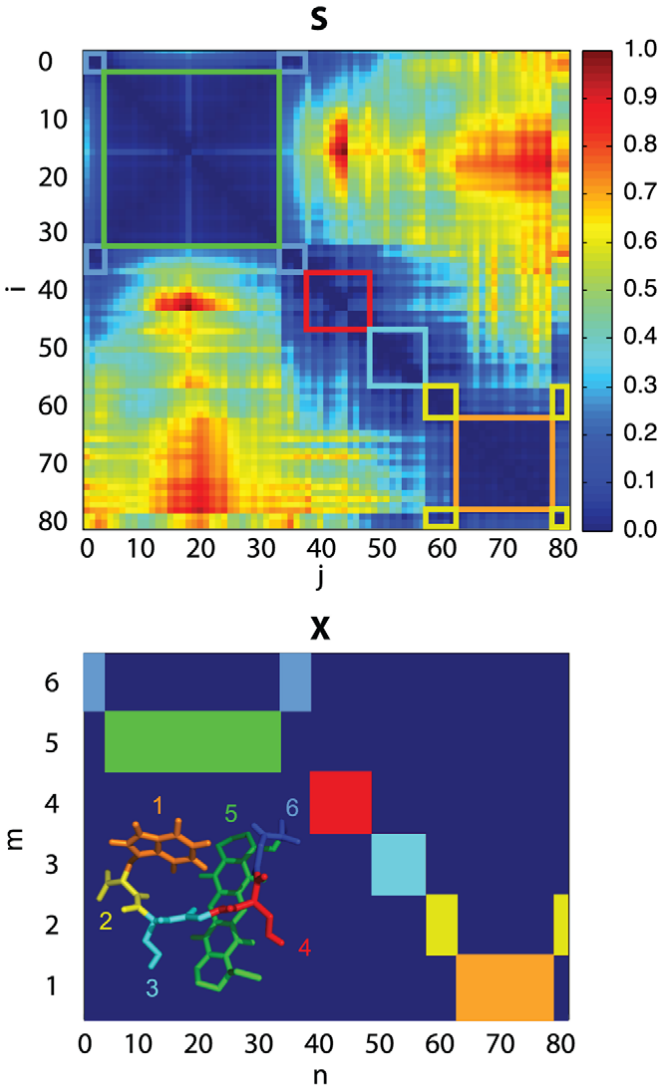
Distance deviation matrix 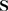 for MR121-GSGSW (top) and membership matrix, 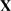, for 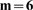 clusters (bottom). The colors relate the semi-rigid regions in the distance deviation matrix to the molecular coarse-grain structure and the membership matrix.

The corresponding distance-deviation matrix is shown in Figure 3 (top). It is structured into blocks along the diagonal (values are close to zero), that represent the almost rigid regions of the molecule. The values on the diagonal are zero (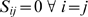) (blue), while values far from the diagonal are large (red). To identify rigid domains within the peptide we have employed the quadratic optimization method for 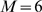. The convergence of the method depends on the size of the molecular system, the number of domains chosen and the initial conditions. For six domains in the artificial peptide (81 atoms) it converged within 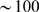 iterations, and a few 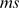 on a standard desktop computer.

The resulting membership matrix, 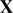, and the corresponding coarse-grain structure are shown in Figure 3 (bottom). The colors show the assignment of atoms to domains. The elements of the membership matrix either converge to one or to zero, i.e. the atoms tilt over to the domain that produces the smallest clustering error when including this atom.

**Figure 4:**
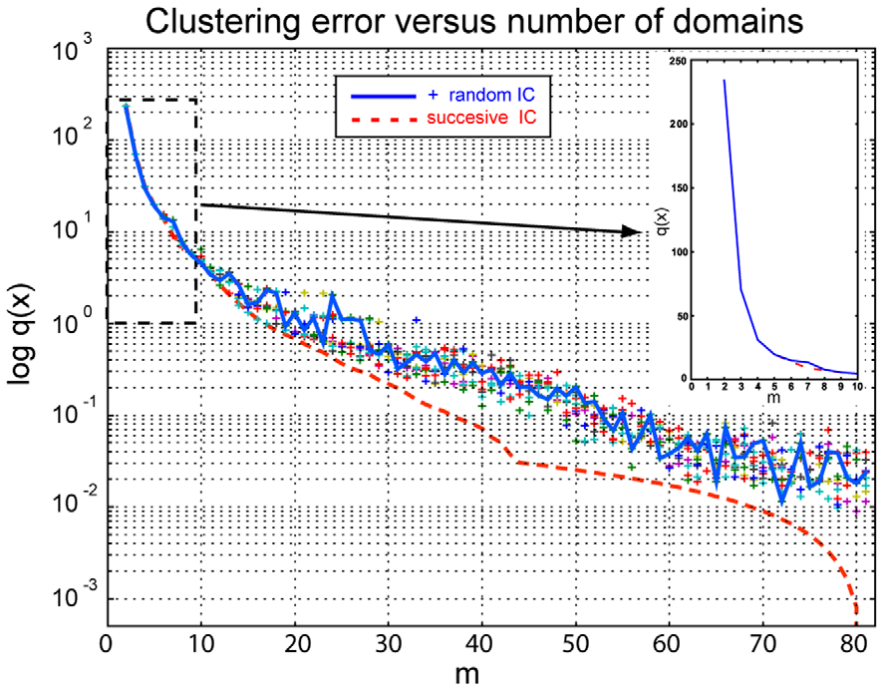
Dependence of clustering error on the choice of the initial condition. When using a random assignment to clusters for the first step 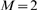 followed by successive restart (dashed red line) the error is monotonically decreasing. Choosing random initial conditions for all 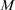 (one realization highlighted as blue solid line, 9 more realizations indicated by “+”), the optimization gets trapped in slightly different local minima for large 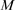. For small 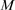 the method robustly identifies the same minimum independent of the initial condition, indicating that global optimality is achieved in this case.

In order to test the optimality of the results, we have repeatedly solved the clustering problem for the MR121-GSGSW peptide using different initial conditions: (i) for given 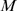, each atom is assigned a random membership to each domain, 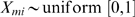 and then normalized so that 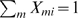; (ii) only 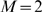 is using a random initial condition while the solutions for 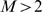 are found by successive restart from the previous solution with the atoms of the largest-error domain split into the two new domains by an initial assignment of 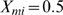. Figure 4 shows a comparison for the clustering error for both cases, with ten realizations for the random initial condition plotted. The results are identical independent of the initial condition for small 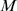, which suggests that these solutions are likely to be globally optimal. For large 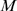 the solution based on random initial condition gets trapped in different but only slightly suboptimal local minima, while the successive restart solution is monotonically improving for increasing 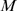. In all cases studied, the heuristic successive restart scheme possesses a useful monotonicity property, and performs better than optimization of random guesses.

### Application to Biological Complexes

#### Transthyretin

**Figure 5:**
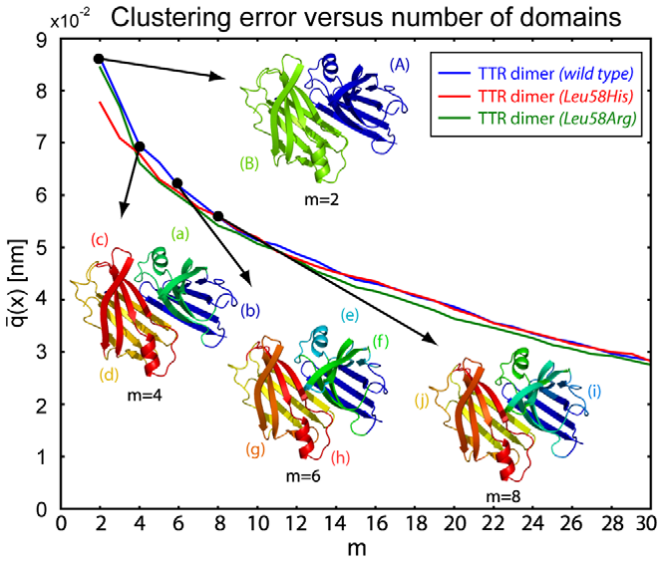
Clustering error of native Transthyretin for 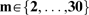 and corresponding coarse-grain structures for 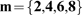 domains. The obtained coarse-grain structures separate the dimer into two monomers (A) and (B) for 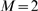 and identify the 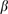-sandwich structure (a)+(b) and (c)+(d) in the two monomers for 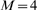. For 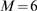 the method additionally identifies the 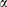-helical structure (e) and (h), for 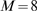 two flexible loops (i) and (j) are found.

The transport protein transthyretin (TTR) is primarily synthesized in liver, choroid plexus, and the retina. The primary function is the transport of thyroxine and retinol binding protein (RBP). Both molecules can bind to the homo-tetrameric structure of TTR, which is found at a physiological pH of 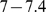. In contrast the 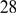 kDa dimer structure is observed at pH 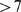 and titration of 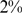 sodium dextransulfate (SDS). It has two identical 127-amino-acid monomers (A - blue) and (B - green) (see Figure 5) with an extensive 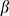-sheet structure that form 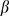-sandwiches [43]. The interactions between the two monomers involve electrostatic and hydrophobic forces.

Transthyretin is one of the human proteins known to be associated with local amyloidosis. Amyloid fibrils are the polymerized form of the protein, their internal structure mainly consists of cross 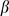-sheets, arranged perpendicular to the long axis of the fibrils [44]. Both point variants of TTR and the native protein are known to deposit as amyloid fibrils in the extra-cellular region, where they cause neurodegeneration and organ failure (for reviews on amyloidosis see [45], [46]). Transthyretin is known to be associated with the amyloid diseases senile systemic amyloidosis (SSA), familial amyloid polyneuropathy (FAP), and familial amyloid cardiomyopathy (FAC). Other known amyloidogenic diseases are for e.g. Alzheimer's disease, type 2 diabetes and the transmissible spongiform encephalopathies which are characterized by proteinaceous deposits in the affected relevant organs.

Transthyretin aggregation to amyloid fibers has been the subject of many studies [43]–[48], however the molecular mechanisms are still not completely understood. Structural modifications and their effect on conformational stability were studied by structural and computational analyses [49] and experimentally by urea and temperature induced unfolding [50], [51]. It is proposed that amyloidogenicity of TTR is associated with anomalous structures that favour oligomer and fibril formation. The structures are assumed to be the product of complex dissociation via destabilisation [52] and subsequent unfolding and folding of the protein [53]. It could be verified that prior fibril formation the homo-tetramer dissociates into two dimers [54]. Whether the dimers need to dissociate into monomers before fibrillation can occur is still unclear. However it is assumed that dimer dissociation is the result of a mechanism called “edge exposure”, where the displacement of residues 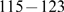 (inner 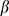-strand) and residues 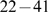 (outer 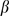-strand) flattens the dimer structure [55], [56].

To date, a large number of TTR variants have been associated with amyloid formation [57]. Here we study the structural rigidity of the wild-type and two variants commonly found, where the leucine of residue 58 in the dimers is replaced by arginine or histidine (TTR-58Arg and TTR-58His) and investigate their possible role in destabilisation and dissociation of the dimer structure. Both variants are known to be amyloidogenic, however the phenotypic difference of FAP between the 58His and 58Arg mutations suggest differences in the secretion efficiency or aggregation characteristics of the TTR variants [58].

In Figure 5 we show the clustering error for increasing number of domains and the coarse-grain structures for 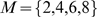 domains. As expected for 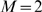 the atoms from each monomer are placed in separate but symmetric domains. This separation is maintained for larger values of 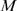. For 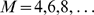 the domains found in the two monomers are nearly, but not perfectly symmetrical, as a result of limited statistical accuracy of the molecular dynamics trajectory. For 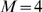 the algorithm identifies two 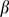-sheets (b) and (d) in the dimer and two structures (a) and (c) including 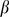-sheets and the 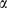-helices. At 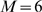 the structures (a) and (c) are split into one block containing two 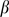-strands (f) and (g) and one block containing two 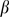-strands and the 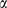-helix (e) and (h). For 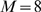 the two loops in the outer region containing two short 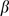-strands are found to be separate domains (i) and (j).

**Figure 6:**
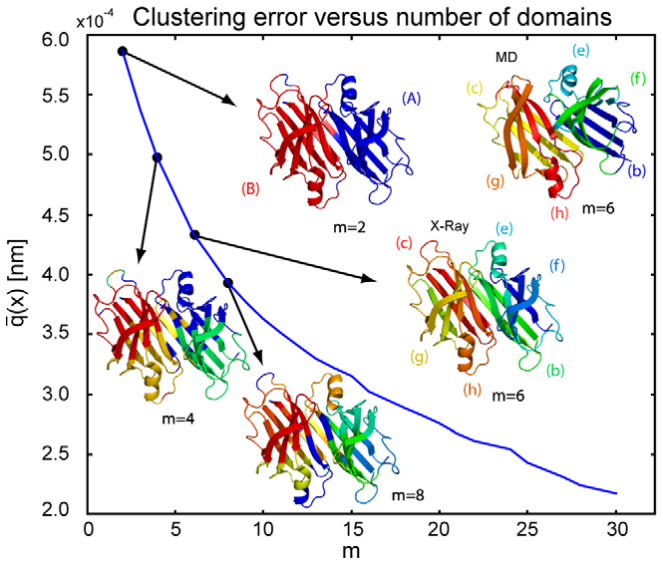
Clustering error obtained from six x-ray structures of Transthyretin for 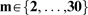 and corresponding coarse-grain structures for 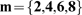 domains. As for molecular dynamics data the obtained coarse-grain structures separate the dimer into two monomers (A) and (B) for 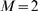 and identify the 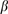-sandwich (b)+(c)+(f)+(g) and 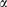-helical (e)+(h) structures in both monomers for 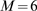.

To demonstrate the applicability to experimental data we used the method to partition molecular structures of transthyretin obtained by x-ray crystallography. Besides the wild type structure (PDB code 1DVQ), which was also used in the molecular dynamics simulation, five related structures of transthyretin complexed with resveratrol, diclofenac, flurbiprofen, DDBF, oFLU, and PHENOX (PDB codes 1DVS, 1DVT, 1DVU, 1DVX, 1DVY, 1DVZ) [39] have been used to generate the distance deviation matrix. Due to in sequence mutations in the structures 1DVX and 1DVZ we cleaned up the structure files to leave only comparable 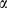-carbon atoms in all six pdb-files. In the 1DVX file we removed residue 9+127 BLEU, 110+228 BSER and 113+231 BTHR, while in 1DVZ 7+124 BLYS and 10+127 BLEU where removed. We note that these six crystallographic x-ray structures correspond to different chemical or crystallographic states, so that the structural differences between them are not expected to be idendical to the structural differences within the Boltzmann-weighted ensemble of a solvated TTR in a single chemical state. Nevertheless, it is expected that the differences in the crystallographic realizations are sensitive to the molecule's instrinsic flexibility, so that a comparison between the simulation-based and X-ray-based results is interesting. The clustering result obtained from the x-ray structures and clustering error with coarse-grain structures for 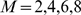 are shown in Figure 6. The clustering of x-ray structures yield similar coarse-graining as obtained by the clustering of molecular dynamics data. The dimer is separated into two monomers (A) and (B) for 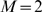, while for 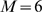 the 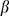-sandwich (b)+(c)+(g)+(f) and 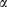-helical (e)+(h) structures within the dimers are identified (compare top right structure generated from MD data). However, for increasing number of domains (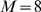) the clusterings are different. Note that the clustering error found for the X-ray structures is much smaller, indicating that the crystallographic realizations are much more similar to each other than the structures accessible to the dynamics of TTR in a solvent simulation, likely owing to crystal lattice constraints.

**Figure 7:**
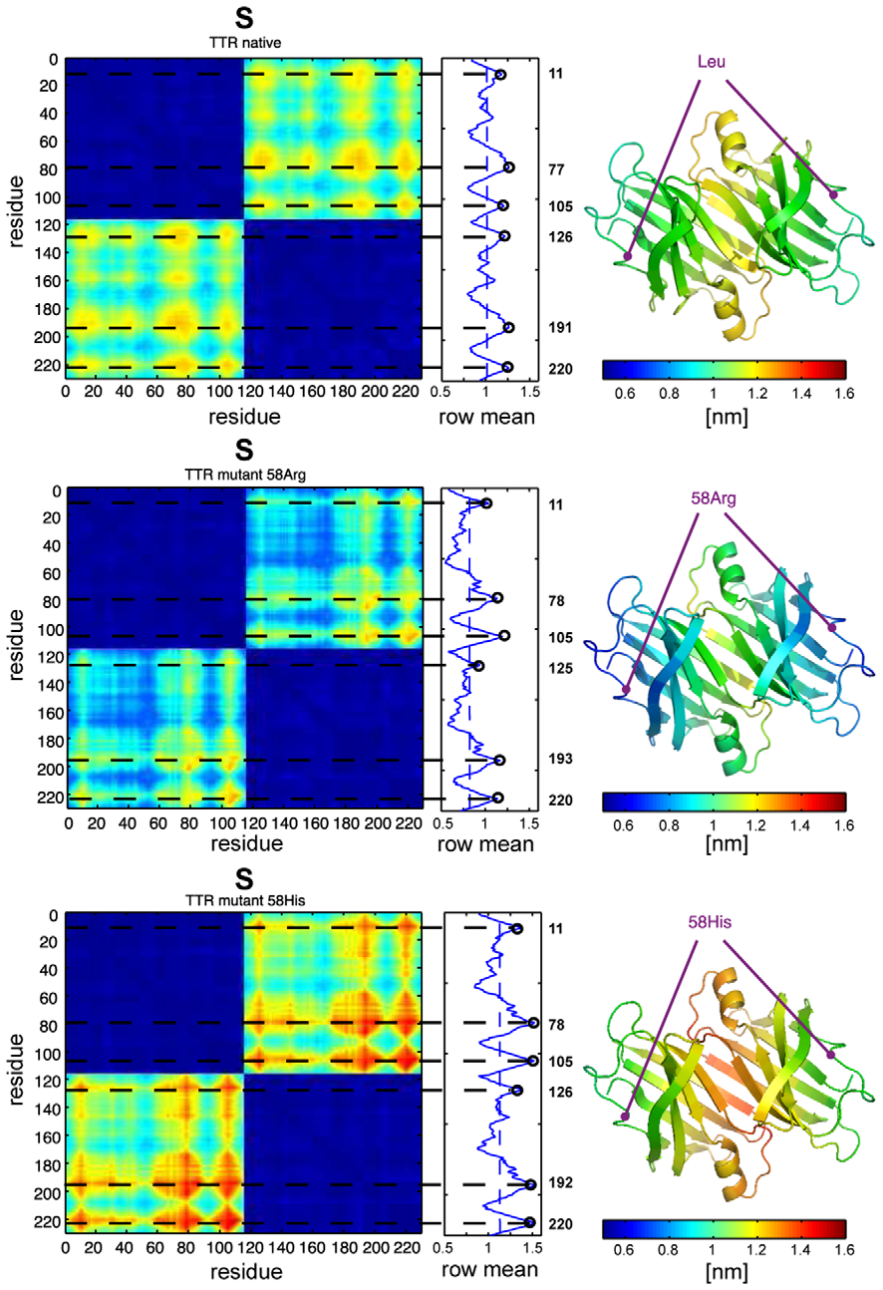
Distance deviation matrix for native TTR (top) compared to variants 58Arg (middle) and 58His (bottom). The mean row value of each matrix indicates flexible regions around reference residues 11, 77, 105, 191 and 220. The corresponding structures are color coded according to the average row value of 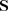 and show the location of residue 58 (purple). Large values (red) indicate flexible regions, while small values (blue) indicate rigid regions in the dimer. The data suggests that the dimer interface is destabilized for both amylogenic TTR variants of the protein.

The distance-deviation matrices in Figure 7 show two large blocks along the diagonal (blue) that indicate that the internal rigidity within each of the two associated monomers is much larger than the rigidity between the monomers. The off-diagonal regions in the matrices (yellow-green-red) represent the inter-monomeric rigidity and are related to the stability or binding strength between the monomers. Large values in the matrix (red) indicate low stability, while small values (blue) are related to high stability of the dimer.

The structural modification induced by the amyloidogenic variants (TTR-58Arg and TTR-58His) contribute to an de/increase in rigidity in some regions of the structure (see increasing red regions in the variants compared to the native TTR in Figure 7), which lead to local de/stabilisation of the dimer. The overall stability of the dimer is directly related to the difference 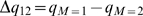. Here, the binding strength 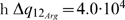 for TTR-58Arg is increased and 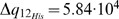 for TTR-58His is decreased compared to the binding strength of the wild-type 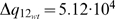. The decreased stability for TTR-58His variant compared to the wild type protein is supported by urea and thermal induced unfolding experiments [51] and computational studies that are based on an energy functions derived from non-redundant x-ray structures [49].

However in addition to the overall stability, increased atomic motion of specific regions in the dimer may influence the stability of the dimer and favor transient dissociation. The local flexibility/rigidity of atoms is reflected by 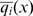, i.e. the mean row value of 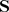. The method is thus able to determine the relevant substructures that may cause destabilisation by taking the row average of the distance deviation matrix (see Figure 7). The peaks indicate residues that have increased distance deviation with respect to all other residues, i.e. the most flexible regions in the dimer. In Figure 7 (right) the structures are color coded according to the row average of 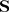, the mutated residue 58 is colored purple. In agreement with [51] the results indicate that compared to the wild-type protein the 58His and 58Arg variants are mainly destabilized at the monomer-monomer interface. In comparison to the wild-type TTR (Figure 7 top), it is clearly seen that the 58His variant increases the total distance deviation between the monomers (see average value of the row mean) and the peak values at residues 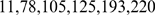. In contrast the 58Arg variant of transthyretin decreases the mean distance deviation, while the peak values at residues 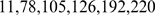 are still increased compared to the wild-type TTR. Because both variants are known to be amylogenic [58], we conclude that destabilisation is not only determined by the overall stability, but also by specific regions that cause local destabilisation of the protein that may lead to transient dissociation into monomers. The method is thus able to provide information about regions of TTR that are destabilized in disease causing variants.

#### GroEL-GroES chaperone complex

**Figure 8:**
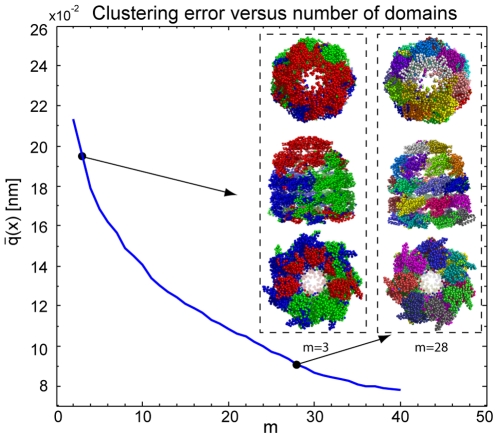
Clustering error for the GroEL-GroES chaperonin complex for 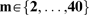 and important structures. The 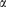-carbon atoms are colored according to the coarse-graining. The poor statistics of the short MD simulation causes discontinuous domains (fragmentation) for small 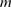. The method clearly detects the functional domains of the complex for 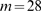.

The existence of semi-rigid domains and their relative dynamics are essential for the functionality of large macromolecular machines. Here, we analyse the dynamics of the GroEL-GroES chaperone complex (see Figure 8), which contains 8,015 residues (72,716 atoms). The complex ensures the proper folding of many proteins [59] and avoids non-native protein aggregation. GroEL is a tetradecameric protein of 14 identical domains arranged in a *cis* and *trans* heptameric-ring. GroES is dome shaped in either un-/bound configuration and contains seven identical domains assembled as a heptamer ring.

**Figure 9:**
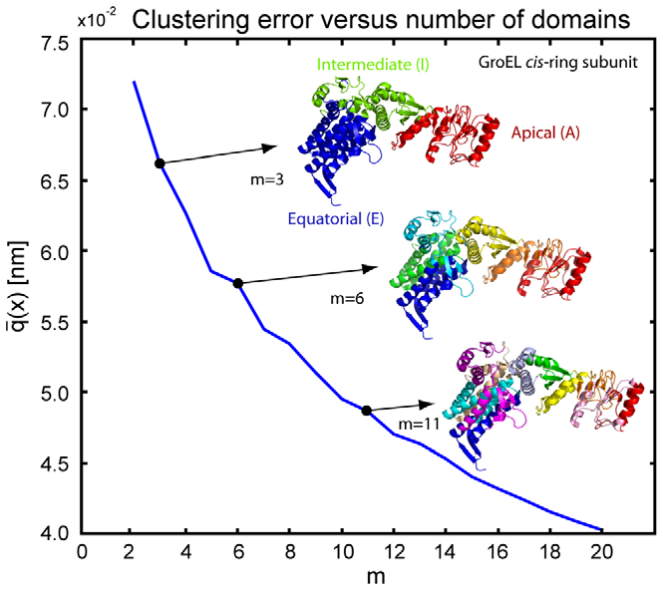
Clustering error for the heptameric subunit of GroEL for 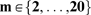. The cartoon representation of three important structures (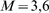 and 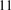) is colored according to the identified domains. For 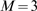 the three functional domains (apical, intermediate and equatorial) in the GroEL subunit are found.

Computational studies have provided important insights into the allosteric mechanism of the chaperonin GroEL-GroES. Protein folding within the complex involves binding, encapsulation, and release of the substrate protein [60], [61]. During the GroEL-GroES cycle the GroEL binds a mis-/unfolded protein at its apical (A) domain (see Figure 9). The binding is caused by electrostatic and hydrophobic interactions between the exposed hydrophobic residues of the substrate protein and those of the apical domain. The equatorial domain (E) plays the major role in the overall chaperonin activity. It binds and hydrolyzes ATP. The intermediate domain (I) serves as a functional bridge between the apical and equatorial domains. After ATP binding to every *cis*-subunit, GroEL is bound to the cofactor GroES. During the GroES binding large conformational changes at the apical domain of the *cis*-ring cause upwards and outwards movement of the apical GroEL domains, thereby increasing the size of the central cavity and forming a dome-shaped chamber [59], [61]. By this conformational change the substrate protein is captured inside the cavity, where it will be able to undergo conformational changes toward the folded state. During ATPs hydrolysis in the *cis*-ring, ATP molecules are transferred to the *trans*-ring, which drives the release of the GroES cap and the substrate protein.

Due to the size of the system only a short molecular dynamics trajectory with duration of 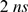 was produced, which is certainly not converged, but can nevertheless be used for a performance test (see Section on “Computational Performance”). In Figure 8 we show the clustering error for 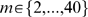 and the most informative structures. We note that due to insufficient statistical information in the MD simulations (under-sampling) the optimization results in disconnected domains (fragmentation) for small 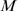 (see Figure 8). Nevertheless, the clustering with 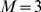 reveals the ring structure of the GroEL into two halves and finds the GroES as a third domain revealing the essential elements necessary to represent the conformational change caused by the complex formation of GroEL with GroES. For a large number of domains, e.g. for 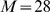, the method clearly detects functional domains that can be directly related to the heptamer-ring structure of the chaperone. All shown results (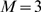, 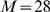) are equally “correct”, but reveal different levels of detail. The fragmentation of domains for small 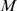 may be reduced by using longer molecular dynamics trajectories. Thus, the method is applicable to large molecular complexes with modest requirements of computation time, but as it is data-based the results are sensitive to the quality of the data.

**Figure 10:**
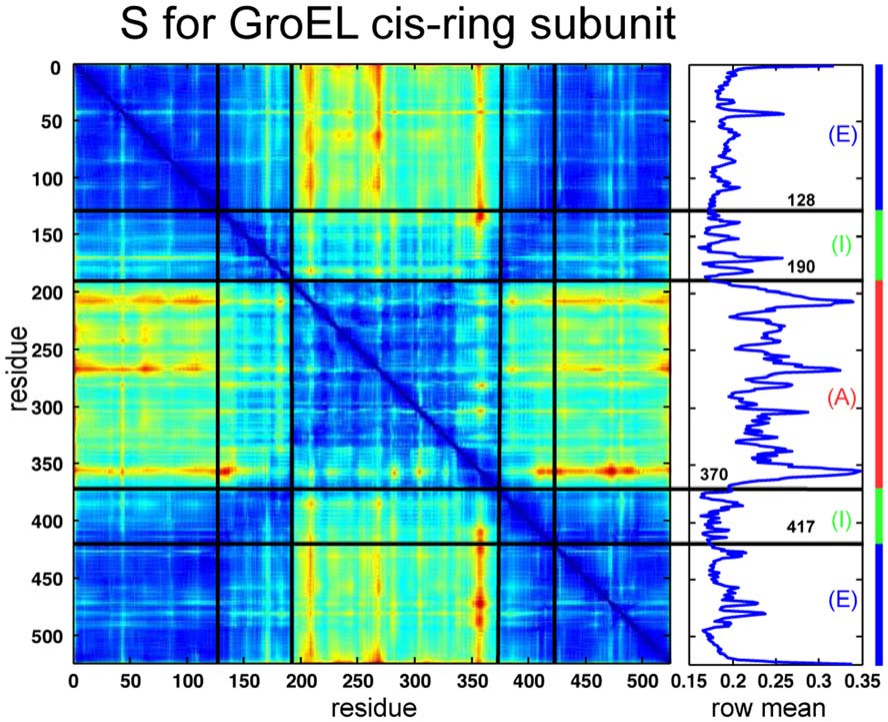
Distance deviation matrix 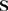 for the heptameric subunit of GroEL structured for 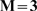. The black lines indicate the identified domains boundaries between the apical (A - red), intermediate (I - green) and equatorial (E - blue) domain in the GroEL subunit. The mean value of each row and the color assignment are shown on the right.

To study GroEL in more detail, we have further performed domain identification on the subunit of the *cis* and *trans* heptameric-rings of GroEL. The distance deviation matrix was generated by averaging over the data of seven identical subunits for the *cis* and *trans* ring respectively. This averaging enhances the statistics of the molecular dynamics data. The domains found in the optimization (see Figure 9) are in good agreement with the functional domains in the GroEL subunit [62]. For 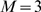 the major three domains (apical, intermediate and equatorial) are found in either *cis*- or *trans*-ring subunits. The distance deviation matrix for 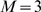 and the identified domain boundaries are shown in Figure 10. The average row value and the color coding for the three domains is shown on the right. These coarse-grain structures are in good agreement to those used in rigid clusters models [63] or Markov models [64]. For larger number of domains, for e.g. 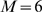, the method identifies two domains in the apical, intermediate and equatorial region respectively. For 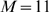 three domains in the apical and intermediate region respectively and four domains in the equatorial region were found.

## Discussion

The coarse-graining algorithm developed in this paper is an optimal and systematic approach to decompose ensembles of molecular structures into semi-rigid domains. It consists of three steps: (i) obtaining an ensemble containing the atomic fluctuations, e.g. using molecular dynamics simulation, (ii) computation of the pair distance-deviation matrix and (iii) definition of semi-rigid domains by a quadratic optimization method, to distinguish and to quantify the rigid and flexible domains within the protein structure. The method identifies rigid regions that can vary in size and shape. The objective function minimized in the procedure is a direct measure of the clustering error and thus the within-cluster flexibility neglected by assigning the atoms into domains. We have been able to study the rigidity of proteins in systems involving 8,015 residues on a normal desktop computer.

In contrast to other methods the algorithm does not require the choice of any parameters other than the number of domains. Being able to fix the number of domains is an advantage, since it gives the user a tool to decide how much flexibility he wants to resolve and to control the magnitude of the clustering error. A straightforward automatic way to select the number of domains is by requiring the clustering error to be below a specified threshold.

The coarse-graining algorithm has been applied to a number of benchmark problems. First, the consistency and error dependence of the method was demonstrated on two short peptides, by systematically increasing the number of domains 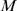 from 2 to the number of atoms 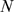. By using appropriate initial conditions, the clustering error was shown to be monotonically decreasing towards zero for 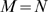. The method was also used to quantify the overall stability/rigidity of several variants of the amyloidogenic protein transthyretin (58His and 58Arg) compared to its native structure. The rigidity properties could be correlated to the destabilisation and amyloid-formation properties of the protein. Compared to the wild type protein we found a decreased stability for TTR-58His variant which is in agreement with urea and thermal induced unfolding experiments of the protein variants [51] and structural and computational studies [49]. It was further found that the TTR destabilisation is not only determined by the overall stability, but also by local destabilisation that is different in the variants. The method is able to identify the residues in the disease causing variants of the protein, that have increased flexibility compared to the wild type protein. These regions are proposed to cause local destabilisation of the protein that may lead to transient dissociation into monomers. For small number of domains the coarse-graining of x-ray structures is almost similar to the coarse-graining obtained by the clustering of molecular dynamics data. Finally, we demonstrated that the rigidity clustering of large molecular complexes like for example the 8,015-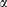-carbon atom system GroEL-GroES can be done within less than one CPU hour. The method clearly identifies functional and structural domains that allow to describe the conformational change of the GroEL-GroES complex formation where the ring structure is split along the long axis resulting in a deformation of the cavity. For larger number of domains the method finds the monomeric substructures in the heptameric rings of the molecular complex. The three major domains found in such a subunit are in good agreement with the apical, intermediate and equatorial domain in the GroEL monomer [62].

Since the clustering method proposed here is a data-based method, its result will depend on the quality of that data. In principle, the result will only be globally converged, if the underlying simulations have visited all relevant conformations within the data set according to the Boltzmann probability. However the GroEL-GroES results and other studies on very large systems such as viruses [65], [66] indicate that the rigidity information required to identify semi-rigid domains within one conformation converges very quickly. The advantage of data-based clustering is that it is independent of the molecular model used and can also be applied to realizations of an NMR ensemble or a series of x-ray structures of the same protein.

Besides the robustness and reliability the method is easy to implement, efficient and useful in obtaining the essential nanomechanical properties of the molecule, we expect it to become a useful tool for the analysis of large-scale molecular systems.

As an outlook the method presented here can be used as a first step to generate a simulation model for large molecules or aggregates that can for example be simulated with Brownian dynamics. In addition to the identification of mobile domains this requires also the estimation of interaction forces and diffusion constants from either simulation or experimental data. This task is a subject of ongoing work.
